# A Two-Step Approach for the Design and Generation of Nanobodies

**DOI:** 10.3390/ijms19113444

**Published:** 2018-11-02

**Authors:** Hanna J. Wagner, Sarah Wehrle, Etienne Weiss, Marco Cavallari, Wilfried Weber

**Affiliations:** 1 Faculty of Biology, University of Freiburg, Schaenzlestraße 1, 79104 Freiburg, Germany; hanna.wagner@biologie.uni-freiburg.de (H.J.W.); sarah.wehrle@epfl.ch (S.W.); 2 Spemann Graduate School of Biology and Medicine (SGBM), University of Freiburg, Albertstraße 19A, 79104 Freiburg, Germany; 3 BIOSS Centre for Biological Signalling Studies, University of Freiburg, Schaenzlestraße 18, 79104 Freiburg, Germany; 4 Ecole Supérieure de Biotechnologie de Strasbourg, UMR 7242, CNRS/Université de Strasbourg, boulevard Sébastien Brant, 67412 Illkirch, France; etienne.weiss@unistra.fr

**Keywords:** complementarity-determining region (CDR) grafting, hapten, toxin, phage display, single-domain antibody, synthetic library, VHH

## Abstract

Nanobodies, the smallest possible antibody format, have become of considerable interest for biotechnological and immunotherapeutic applications. They show excellent robustness, are non-immunogenic in humans, and can easily be engineered and produced in prokaryotic hosts. Traditionally, nanobodies are selected from camelid immune libraries involving the maintenance and treatment of animals. Recent advances have involved the generation of nanobodies from naïve or synthetic libraries. However, such approaches demand large library sizes and sophisticated selection procedures. Here, we propose an alternative, two-step approach for the design and generation of nanobodies. In a first step, complementarity-determining regions (CDRs) are grafted from conventional antibody formats onto nanobody frameworks, generating weak antigen binders. In a second step, the weak binders serve as templates to design focused synthetic phage libraries for affinity maturation. We validated this approach by grafting toxin- and hapten-specific CDRs onto frameworks derived from variable domains of camelid heavy-chain-only antibodies (VHH). We then affinity matured the hapten binder via panning of a synthetic phage library. We suggest that this strategy can complement existing immune, naïve, and synthetic library based methods, requiring neither animal experiments, nor large libraries, nor sophisticated selection protocols.

## 1. Introduction

The FDA approval of the first monoclonal antibody, Orthoclone OKT3^TM^, for clinical use in 1985 marked a new era in the therapeutic sector, opening the door for novel cancer, autoimmune, and viral disease treatments [1,2,3]. With ten new antibody therapeutics making their way to the market in 2017, the annual number of approvals by the FDA and the EMA reached the double-digit level [4]. However, antibodies intended for diagnostic or therapeutic use are required to fulfill stringent criteria to ensure their functional performance and safe administration. These include low immunogenicity, high tissue penetration, and excellent stability, in addition to cost-efficient production at high yields. Although full-length antibodies are conventionally administered, they do not fulfill all those criteria. Unless human or humanized antibodies are used, they are prone to provoking immune responses in the patient, which can lead to ineffectiveness or worse, adverse effects. The size of full-length antibodies (~150 kDa) prevents deep tissue penetration and thus efficient reaching of target sites. Furthermore, the structure of antibodies contains intra- and interchain disulfide bridges as well as glycosylations [5], which is crucial for their effectiveness but limits their production to eukaryotic hosts. Consequently, alternative antibody formats are currently under intensive investigation. Several artificial affinity proteins—such as affibodies [6,7], monobodies [8], or anticalins [9]—fulfill the abovementioned requirements of therapeutic antibodies. However, their development is far from trivial and necessitates sophisticated synthetic libraries and selection methods. Moreover, their small size and structure limit their specificity mainly to larger, proteinaceous antigens [10]. Single-domain antibodies (sdAbs), on the other hand, can classically be selected from immune libraries—binders against both proteins and haptens have been discovered [11,12]. These small antibody fragments (~15 kDa), also known as nanobodies [13], are derived from the variable domain of heavy-chain-only antibodies (HCAbs), found in the serum of camelids (VHH) or cartilaginous fishes (VNAR) [14]. Nanobodies are characterized by high thermal stability and solubility, unique refolding properties, and high production yields in bacteria or other hosts [15]. They exhibit affinities comparable to conventional antibodies and superior tissue penetration due to their small size. Because of these favorable characteristics, nanobodies are suitable candidates for the development of imaging probes [16,17], therapeutic agents with neutralizing or receptor-ligand antagonizing functions [18], and for targeted drug therapy [19,20]. Recently, a nanobody-based treatment (Cablivi) for acquired thrombotic thrombocytopenic purpura was approved in Europe [21,22]. Moreover, nanobodies are used for analytical purposes and offer broad applications in research, for example in affinity chromatography [23] or chromatin immunoprecipitation [24].

Conventionally, nanobodies are selected from immune phage libraries [25,26,27]. Until today, a variety of VHH with specificities against different classes of molecules such as protein ligands [28], hormones [29,30], small molecule drugs [31], toxins [32,33,34,35], and other chemicals [36,37] have been developed. However, immunization is a time-consuming process and requires the costly maintenance of large animals, for example camelids. This can be circumvented by alternative methods, such as the use of synthetic libraries [38] that have been successfully applied for the selection of nanobodies against different protein targets and even resulted in conformation-specific nanobodies and intrabodies [39]. However, working with such libraries that are not pre-enriched for the target requires a large library size or subtle selection strategies making the generation of binders to small molecules such as haptens a challenging task [40].

Here, we present an alternative method for the generation of VHH that involves the grafting of the complementarity determining regions (CDRs) from already existing, non-camelid antibodies to VHH frameworks, followed by affinity maturation using synthetic phage libraries. To date, myriad antibodies and fragments thereof have been generated against manifold antigens. This existing assortment represents an ideal CDR donor repertoire for our approach. CDR grafting is a powerful technique for transferring binding specificities to other antibody frameworks with desired properties. This is typically done to stabilize or humanize antibodies intended for medical use [41,42,43].

In the context of nanobodies, universal scaffolds have been identified, enabling the generation of robust or humanized VHH variants [44,45,46]. So far, this strategy has only been applied to graft CDRs to acceptor frames obtained from animals of the same taxonomic family. The rational design of nanobodies based on CDRs derived from conventional, pre-existing antibodies would be of high value to avoid animal immunizations and directly generate binders in the desired framework. However, the design of nanobody grafts with CDRs derived from conventional antibodies requires careful consideration, because both the heavy and light chain variable domain (VH and VL) form the antigen binding site and are involved in the recognition of the antigenic epitope. Furthermore, the framework plays an important role in CDR conformation and orientation and distinct framework residues often contribute directly to antigen binding. Therefore, framework residues always have to be considered in the design of the graft [43,47]. 

**Figure 1:**
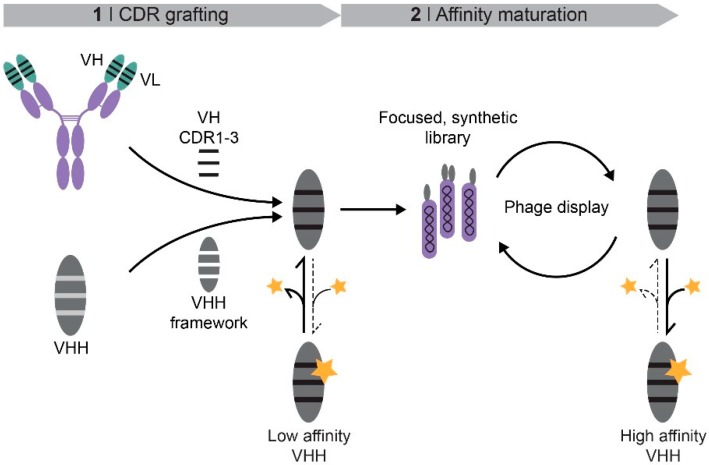
Schematic overview of a two-step approach for the generation of VHH. First, the complementary-determining regions (CDRs) of the heavy-chain variable domain (VH) of a conventional antibody are grafted onto a VHH framework. In a second step, the resulting weak binders, which lack the light-chain variable domain (VL), serve as template for the design of a focused VHH library. The low association rate with the ligand (yellow star) is indicated by the dotted line, the dissociation by the solid line. Affinity maturation is conducted by phage display.

Here, we propose the grafting of CDRs from pre-existing antibodies of non-camelid origin to VHH frameworks as the basis for the generation of nanobodies (Figure 1). Since the rational design of chimeric nanobodies involving CDR donors and acceptors of different taxonomic orders may not always be sufficient, the resulting grafted constructs may serve as templates for further affinity maturation by panning of synthetic phage libraries (Figure 1). Employing this CDR grafting approach, we obtained weak protein- and hapten-binding nanobodies. In a second step, we generated a synthetic phage library based on the hapten-binding VHH and selected a binder with nanomolar affinity. Based on these results, we suggest that this strategy has general applicability and provides a versatile alternative to protocols involving immunizations or sophisticated selection methods.

## 2. Results

### 2.1. Design of Toxin- and Hapten-Specific VHH Grafts

**Figure 2:**
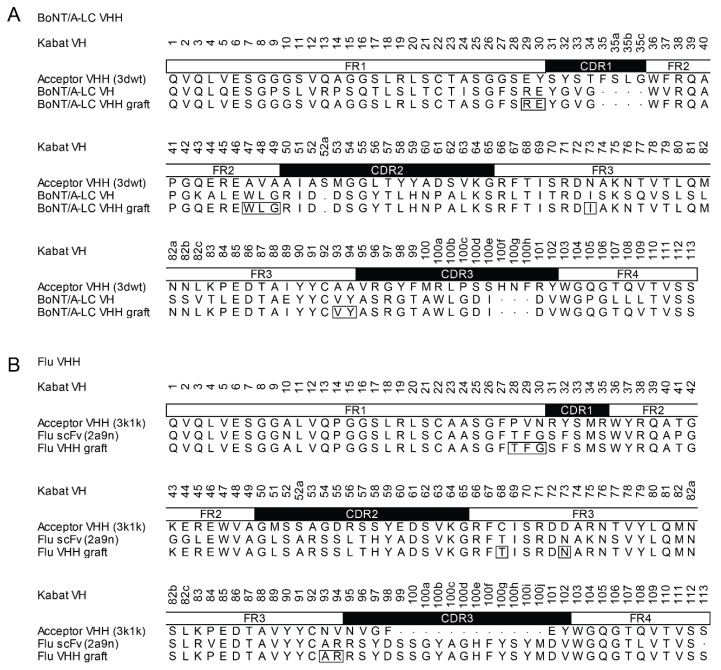
Sequence alignments of the complementarity determining region (CDR) donor VH, the acceptor VHH and the grafted VHH. (**A**) Design of a BoNT/A-LC-binding VHH. VH-CDRs of the BoNT/A-LC-binding scFv (FJ643069 [48]) were grafted onto the framework of the cAbBCII-10 nanobody (3dwt [45]). (**B**) CDR-grafting of a fluorescein-specific VHH. VH-CDRs of the Flu-scFv-E2 (2a9n [49]) were grafted onto the framework of the enhancer nanobody (3k1k [50]). Numbering and CDR definition are according to Kabat et al. [51]. Framework residues corresponding to the upper core of the framework [47,52] were matched according to the donor sequence and are marked by white boxes.

In this study, we evaluated a two-step design approach for the generation of VHH involving the grafting of CDR from non-camelid donors to VHH frameworks, followed by affinity maturation using a synthetic phage library. As model antigens, we chose the Botulinum neurotoxin A (BoNT/A) and the small molecule fluorescein. Antibodies and antibody fragments against both targets have been generated, and we used the VH domains of scFvs binding to the light chain of BoNT (BoNT/A-LC; FJ643069 [48]) or to fluorescein (2a9n [49]) as CDR donors for our designs. We evaluated the cAbBCII-10 nanobody (3dwt [45]) and the enhancer nanobody (3k1k [50]) as acceptors for the BoNT/A-LC and fluorescein-specific CDRs, respectively. Figure 2 shows the sequence alignments of the constructs. CDRs and framework regions were identified according to the Kabat numbering scheme [51]. In addition to the CDRs, residues in the upper core of the variable domains affecting CDR conformation and orientation were matched according to the donor sequences [43,47,52]. The resulting changes in the frameworks are marked by boxes in Figure 2.

### 2.2. Evaluation of Antigen Binding

**Figure 3:**
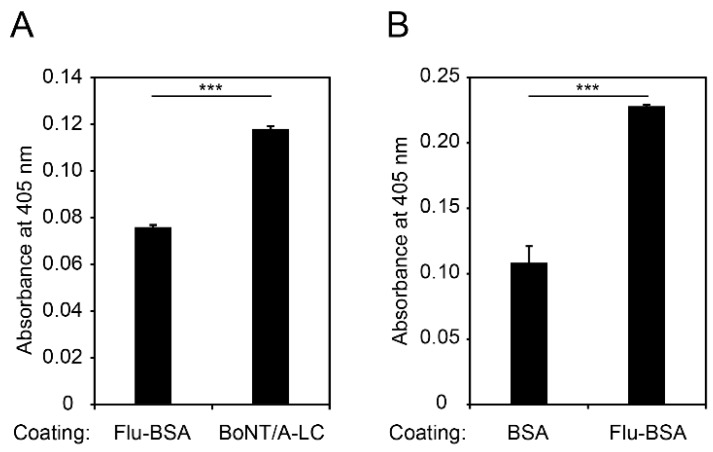
Binding capabilities of BoNT/A-LC and fluorescein-binding VHH grafts. (**A**) ELISA of the BoNT/A-LC-specific VHH graft. Wells were coated with fluorescein-conjugated BSA (negative control) or BoNT/A-LC and the binding of VHH (5 µg/mL) was assessed. (**B**) ELISA of the fluorescein-specific VHH graft. Wells were coated with BSA (negative control) or fluorescein-conjugated BSA and the binding of VHH (100 µg mL^−1^) was evaluated. Mean values of three replicates ± SEM are shown. Statistical analysis: unpaired *t*-test, *** *p* < 0.001.

The grafted VHH were produced in *E. coli*, purified via affinity chromatography (Supplementary Figure S1), and their antigen binding capacity was analyzed by ELISA. As depicted in Figure 3A, the grafted BoNT/A-LC VHH showed slightly stronger binding to its antigen compared to the negative control (fluorescein-conjugated BSA). We obtained similar results for the grafted fluorescein-specific VHH, which bound stronger to fluorescein-conjugated BSA compared to non-conjugated BSA (Figure 3B). Noteworthily, these results were obtained with high VHH concentrations (5 µg mL^−1^ for the BoNT/A-LC and 100 µg mL^−1^ for the fluorescein binder), indicating weak VHH–antigen interactions. Nevertheless, we hypothesized that, similar to immune libraries, sequence randomization of weak binders might enrich binders with higher affinity, mimicking an *in vivo* germinal center reaction. We tested this hypothesis by designing a synthetic VHH library for affinity maturation via phage display. To the best of our knowledge, synthetic libraries have been successfully used for the selection of protein binders [39], but not for hapten-binding VHH. Therefore, we chose the fluorescein-binding VHH for the design of our synthetic library.

### 2.3. Design of a Synthetic VHH Library

**Figure 4:**
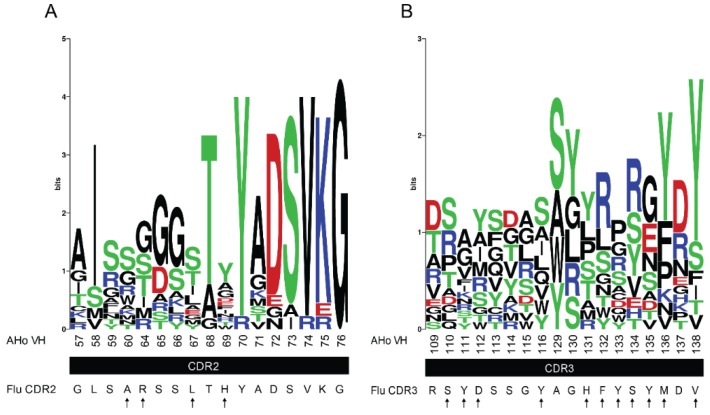
CDR2 and CDR3 sequence logos of protein- and hapten-specific VHH. Eight hapten- and nine protein-binding VHH were aligned to generate sequence logos of CDR2 (**A**) and CDR3 (**B**). The fluorescein-specific CDR sequences are shown below the graphs. The numbering scheme is according to AHo [54]. Residues that were chosen for randomization are marked by arrows. The sequence logos were generated with WebLogo [53].

We designed our synthetic VHH library mainly focusing on CDR3, which is usually the most diverse and important antigen-binding loop in VHH. Additionally, we randomized residues in CDR2. We systematically compared the corresponding amino acids with sequence logos [53] generated by aligning eight hapten- and nine protein-binding VHH (Figure 4). For the alignments, we applied the AHo numbering scheme [54], which minimizes deviations from averaged structures by placing gaps accordingly. Using these sequence logos, we identified positions in CDR2 and CDR3 that show high diversity (such as residues 60, 67 and 69 in CDR2; highlighted by arrows in Figure 4A). Based on this natural diversity, we assumed that these positions are permissive for affinity maturation. Furthermore, we identified positions where abundant amino acids differ in their side chain properties from the fluorescein-specific CDR2 and CDR3. For example, tyrosine is the dominant amino acid at positions 136 and 138 in the (CDR3) sequence logo (Figure 4B) whereas the fluorescein-specific CDR3 contains a methionine and a valine at these positions. We randomized all these positions (highlighted by arrows in Figure 4A,B) by using ambiguous codons for amino acid sets that included the original fluorescein-specific residues and provided residues with similar properties as in the sequence logo and/or amino acids that are highly abundant in antigen-binding loops (mainly tyrosine and serine [55]).

**Figure 5:**
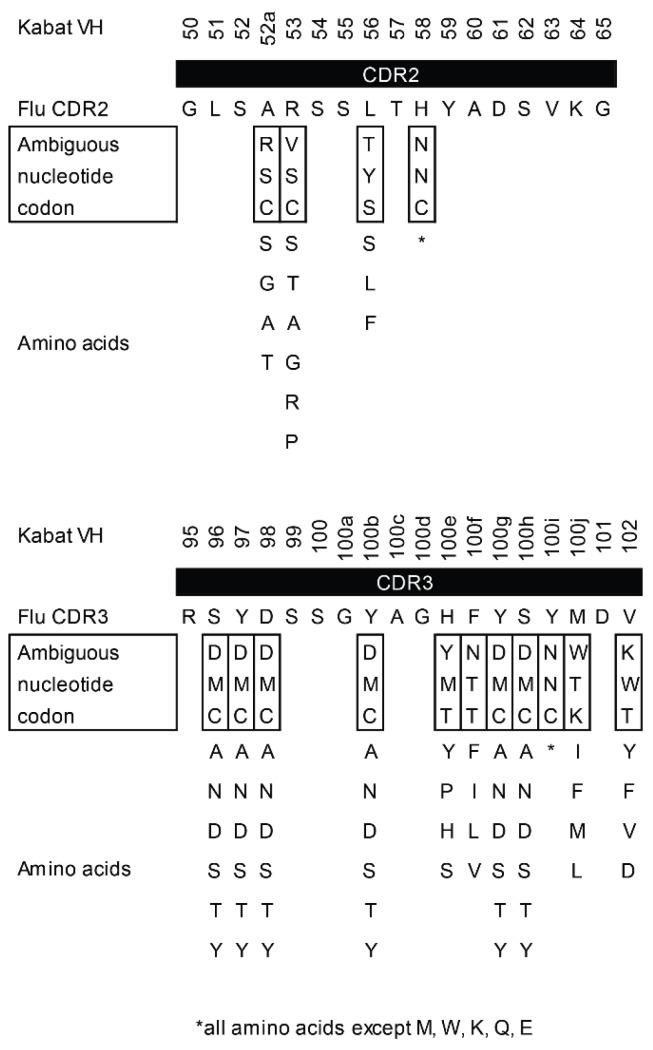
Design of a fluorescein-specific VHH library. CDR2 and CDR3 were randomized using PCR primers containing the indicated ambiguous codons. Nucleotide ambiguities are represented in accordance with the IUPAC code (D = A/G/T, K = G/T, M = A/C, N = A/C/G/T, R = A/G, S = G/C, V = A/C/G, W = A/T, Y = C/T). The corresponding amino acids are shown below the codons. For each position, a codon was used that provided the original amino acid along with residues according to the sequence logo and/or polar amino acids commonly found in CDR regions (tyrosine, serine). Stop codons were excluded from the library design.

Residue 58 (residue 69 according to the AHo numbering scheme) shows the highest diversity in CDR2, however, the sequence logo did not comprise a histidine at this position. Therefore, we used the NNC-codon at this position providing 16 amino acids (all amino acids except for methionine, tryptophan, lysine, glutamine and glutamate). We used the same codon at position 100i (135 according to AHo). Furthermore, we avoided stop codons in our sequence. Figure 5 gives an overview of the assigned ambiguous codons and corresponding amino acids for the target positions in CDR2 and CDR3. This library design represents a focused, fluorescein-specific approach to mature the affinity of our grafted VHH.

### 2.4. Selection of Fluorescein-Binding VHH from the Synthetic Library

**Figure 6:**
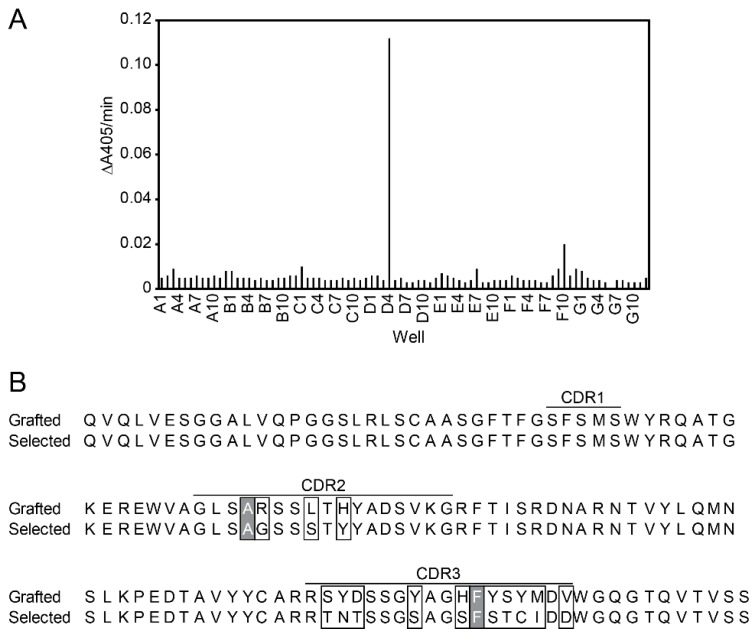
Affinity maturation of fluorescein-binding VHH. (**A**) ELISA screening of fluorescein binders. After two rounds of panning, fluorescein-binding VHH were expressed in *E. coli* and screened for their binding capability to fluorescein-conjugated BSA. (**B**) Sequence alignment of the grafted VHH and the selected VHH-D4. Thirteen out of fifteen randomized residues in CDR2 and CDR3 differ (white boxes). Alanine and phenylalanine in CDR2 and CDR3, respectively, remained unaltered (grey boxes).

We applied a two-step panning approach for the selection of fluorescein-specific VHH. In the first round of panning, we enriched the library for binders using immobilized fluorescein-conjugated BSA and by elution with trypsin. In the second round, we first eluted competitively with 10 µM, then with 1 mM fluorescein and obtained a 100- and 75-fold enrichment of phages eluted from selection versus BSA-control wells, respectively. We screened for binders by ELISA and identified a hit in constructs eluted with 1 mM fluorescein (clone D4 in Figure 6A). Sequence comparison with the grafted fluorescein-specific VHH revealed changes in thirteen out of fifteen possible amino acid positions (white boxes in Figure 6B). Alanine in CDR2 and phenylalanine in CDR3 remained unchanged (grey boxes in Figure 6B).

### 2.5. Characterization of a Selected Fluorescein-Binding VHH

**Figure 7:**
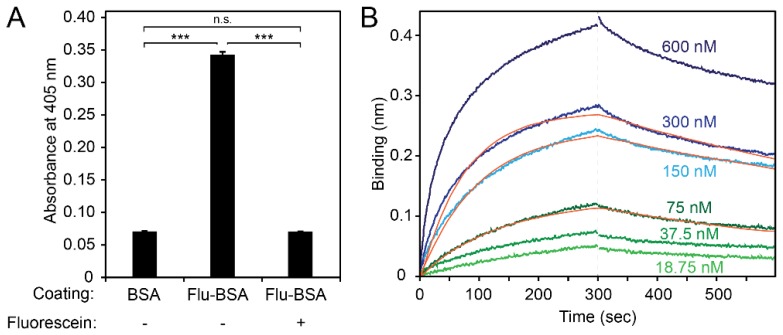
Fluorescein binding of the selected VHH-D4. (**A**) Binding specificity of VHH-D4. Wells were coated with BSA (negative control) or fluorescein-conjugated BSA and the binding of VHH (0.75 µg mL^−1^) was evaluated in the absence (−) or presence (+) of 1 mM free fluorescein. Mean values of three replicates ± SEM are shown. Statistical analysis: one-way ANOVA followed by Tukey’s multiple comparison test; *** *p* < 0.001; n.s., not significant. (**B**) Binding affinity of VHH-D4. Bio-layer interferometry sensorgram traces of the association and dissociation of VHH-D4 to/from fluorescein-conjugated BSA. Aminopropylsilane biosensors were loaded with fluorescein-BSA and association and dissociation of VHH-D4 at the indicated concentrations were monitored. The orange curves represent the global fitting according to a 1:1 bimolecular interaction.

We expressed the selected fluorescein-specific VHH-D4 in *E. coli* (Supplementary Figure S2) and analyzed its binding capacity to fluorescein by ELISA (Figure 7A; Supplementary Figure S3). The VHH-D4 bound to fluorescein-conjugated BSA, but not to BSA. The addition of free fluorescein competed with BSA-conjugated fluorescein in binding to the VHH-D4 and resulted in an ELISA signal comparable to the negative control (BSA coating), demonstrating the capability of our selected nanobody to bind to both conjugated and free, soluble fluorescein.

Next, we evaluated the binding properties of the VHH-D4 by bio-layer interferometry (Figure 7B). We coated aminopropylsilane biosensors with fluorescein-conjugated BSA or BSA (negative control). The highest applied VHH-D4 concentration (600 nM) did not bind to BSA, indicating specificity to its ligand fluorescein. Due to the fluorescence of fluorescein, we observed an increased background signal with Flu-BSA-coated sensors. We subtracted this background signal from the binding and association curves. We obtained k_on_ and k_off_ values of 6.0 × 10^4^ M^−1^s^−1^ and 1.2 × 10^−3^ s^−1^, respectively, resulting in a dissociation constant K_D_ of 20 nM.

These results validate our strategy of grafting antigen-binding loops from conventional antibodies to VHH frames to obtain weak binders whose affinity can be matured by rationally designed phage libraries. We obtained a VHH that bound with affinity in the nanomolar range to fluorescein, suggesting that our rational approach enables the generation of hapten-binding VHH, usually selected from immune libraries.

## 3. Discussion

Methods for the discovery of antibody fragments that involve neither time-consuming nor expensive animal treatments nor elaborate affinity maturation processes are of high interest.

Naïve antibody libraries are constructed from immunoglobulin V gene segments of peripheral blood lymphocytes isolated from non-immunized donors, thus reducing animal treatments to a minimum and representing an alternative to traditional immune libraries [49,56]. Naïve VHH libraries have been used for the isolation of hapten-specific or -mimetic nanobodies [40,57,58]. However, such naïve libraries usually require very large sizes to ensure the selection of high-affinity antibodies [49,59].

In comparison to libraries derived from natural repertoires, synthetic libraries have attracted the attention of antibody engineers because they are neither restricted by the immune system’s self-tolerance nor by the toxicity of some antigens. They also offer the possibility of selecting frameworks with respect to stability, immunogenicity, and expression yield. Furthermore and in contrast to immune libraries, single synthetic libraries can be used for the isolation of antibodies with different specificities. However, the design of synthetic libraries requires detailed knowledge of antibody structure and function. Sophisticated strategies have been applied for the construction of synthetic Fab and scFv libraries [60,61,62,63,64].

In recent years, synthetic libraries have also been reported for VHH. In an initial approach, Yan et al. randomized CDR3 by 16 NNK codons and obtained a ~20-fold enrichment after six rounds of panning against human prealbumin- and neutrophil gelatinase-associated lipocalin [38]. Although the isolated prealbumin-binding VHH showed inferior binding and thermal stability compared to VHH from an immune library, this study demonstrated the feasibility of isolating binders from synthetic VHH libraries. More recently, Moutel et al. constructed a universal synthetic phage display library based on a well-conceived concept [39]. They generated a novel, humanized, and stable scaffold consensus and randomized all three CDR regions with sets of amino acids partially recapitulating natural diversity. Additionally, CDR3 was varied in length. Several protein-binding VHH, including conformation- and tumor antigen-specific binders as well as functional intrabodies, were isolated from this library [39]. McMahon et al. reported a similar approach based on a yeast surface display platform [65].

Similar to naïve libraries, these libraries are not pre-enriched for antigen-binders and thus require large sizes (10^8^–10^9^ individual clones) [38,39,65]. Furthermore, the isolation of small molecule-binding VHH from synthetic libraries has not been reported so far. This may be due to the unique mode of binding of VHH. Their CDR3 loop is usually extended and, together with CDR1 and CDR2, forms a convex paratope that typically reaches into protein clefts or into protein–protein interfaces [14]. Thus, VHH specific for small epitopes are rarely found [15].

In the case of scFv, it has been shown that different types and sizes of antigens (proteins, peptides, carbohydrates, or haptens) require different topographies of the paratope [66]. Persson et al. have exploited this correlation between paratope structure and type of antigen for the development of a focused antibody library biased for hapten-binding scFv [67]. Here, we applied a similarly focused approach for the synthetic generation of small molecule-binding VHH. We grafted CDRs from existing human and sheep antibodies onto VHH frameworks. The change from the two-domain scFv to the single-domain VHH format resulted in an expected loss of affinity. However, we hypothesized that these weak binders represent ideal templates for the design of focused synthetic libraries. We validated this approach by the example of a VHH binding to the small molecule fluorescein. We obtained one hit after the second round of panning. Further rounds of panning or selection processes involving only competitive elution [36] would likely yield additional hits. Out of the thirteen residues that had changed compared to the grafted construct, seven were either tyrosine or serine. This finding is in agreement with the notion that the hydrophilic side chains of these two amino acids are particularly advantageous for molecular recognition [55], and could facilitate the design of synthetic VHH libraries.

The isolated VHH-D4 clone bound specifically and with nanomolar affinity to fluorescein and thus supported the convenience of our two-step approach for generating functional VHH, even against more difficult targets such as small molecules.

We believe that this approach complements immune, naïve, and synthetic libraries for the generation of protein as well as hapten binders. Conveniently, the number of existing antibodies provides a huge repository of CDRs for the design of focused synthetic VHH libraries. Such methods that facilitate the development of VHH with the desired specificities and frameworks represent valuable approaches to advance nanobody-based research and the medical potential of VHH-based therapeutics.

## 4. Materials and Methods

### 4.1. Graft Construction

The designed VHH amino acid sequences were converted to nucleotide sequences with *E. coli* codon usage for bacterial protein production. The VHH-Flu graft was ordered as gBlocks Gene Fragments (Integrated DNA Technologies, Skokie, IL, USA). The VHH-BoNT/A graft was constructed by assembly PCR. Details on plasmid cloning are summarized in Supplementary Tables S1 and S2.

### 4.2. Protein Production and Purification

VHH were produced in *E. coli* BL21(DE3) SHuffle T7 Express (New England Biolabs, Ipswich, MA, USA, #C3029). Strep-tag-BoNT/A-LC was produced in *E. coli* BL21(DE3) pLysS (Thermo Fisher Scientific, Waltham, MA, USA, # C602003). Cells were grown in LB medium, supplemented with 100 µg/mL ampicillin, and in the case of Strep-tag-BoNT/A-LC, additionally with 36 µg/mL chloramphenicol, in shake flasks at 37 °C and 150 rpm. At OD_600_ = 0.6–0.8, protein production was induced by 1 mM isopropyl β-D-1-thiogalactopyranoside (IPTG) at 30 °C for 4 h (VHH) or 25 °C overnight (Strep-tag-BoNT/A-LC). Bacteria were harvested at 6000× *g* for 10 min and resuspended in 35 mL Ni Lysis buffer (50 mM NaH_2_PO_4_, 300 mM NaCl, 10 mM imidazole, pH 8.0). The cells were disrupted by sonication at 60% amplitude and 0.5 sec/1 sec pulse/pause intervals for 10 min on ice (Bandelin Sonoplus HD 3100 homogenizer, BANDELIN, Berlin, Germany) and centrifuged at 30,000× *g* for 30 min at 4 °C. Soluble VHH in the supernatant was either purified via Ni-NTA (HJW268 and HJW206; QIAGEN, Hilden, Germany, #30230) or protein A (HJW202; Roche, Basel, Switzerland, #000000011134515001) affinity chromatography. The cleared lysates were applied to gravity-flow columns (2 mL bed volume), equilibrated with Ni Lysis buffer. The columns were washed twice with Ni wash buffer (Ni-NTA; 50 mM NaH_2_PO_4_, 300 mM NaCl, 20 mM imidazole, pH 8.0) or once with 15 mL PBS (protein A). Proteins on the Ni-NTA material were eluted with 8 mL Ni elution buffer (50 mM NaH_2_PO_4_, 300 mM NaCl, 250 mM imidazole, pH 8.0). Protein A-bound VHH was eluted with 0.1 M glycine, pH 3.0. The pH was neutralized with 1 M Tris-HCl, pH 8.0. Strep-tag-BoNT/A-LC was purified via Strep-Tactin^®^XT (IBA Lifesciences, Göttingen, Germany, #2-4030-002) using gravity flow columns (1 mL bed volume) and equilibrated with Strep-buffer (100 mM Tris-HCl, 150 mM NaCl, pH 8.0). After washing with 5 mL Strep-buffer, the protein was eluted with 3 mL Strep-buffer supplemented with 50 mM biotin.

### 4.3. Synthesis of Flu-BSA

10 mg/mL bovine serum albumin (BSA; Sigma Aldrich, St. Louis, MO, USA, #05479) was incubated with a 20-fold molar excess of fluorescein isothiocyanate (FITC; Sigma Aldrich, #F7250) at RT for 2 h. Uncoupled FITC was removed by gel filtration using a dextran desalting column (5K MWCO; Thermo Fisher Scientific, #43233), followed by dialysis in a SnakeSkin dialysis tubing (3.5K MWCO, Thermo Fisher, #68035) against PBS.

### 4.4. Analytical Methods

Protein concentrations were determined by Bradford assay (Bio-Rad, Hercules, CA, USA, #5000006) using a dilution series of BSA as a protein standard. For evaluating protein production and purification, sodium dodecyl sulfate (SDS)-polyacrylamide gel electrophoresis (SDS-PAGE) was conducted using 12 or 15% (*w*/*v*) SDS-gels, followed by Coomassie staining.

Antigen-binding was analyzed by ELISA. ELISA plates (Corning, Corning, NY, USA, #CORN3590) were coated with 1 µg Flu-BSA, 5 µg BoNT/A-LC, or 1 µg BSA (negative control) per well at RT overnight. The wells were washed three times with 300 µL PBS supplemented with 0.05% (*v*/*v*) Tween-20 (PBST) and blocked with 300 µL PBST supplemented with 1% (*w*/*v*) BSA (blocking buffer) for 1 h at RT. After three washing steps with PBST, a 100 µL sample was added and incubated for 1 h at RT. The wells were washed three times and probed with 100 µL primary anti-His antibody (0.2 µg/mL in blocking buffer; VWR, #NOVG70796-3) for 1 h at RT. After washing three times, 100 µL secondary anti-mouse IgG-HRP (1:2500 in blocking buffer; GE Healthcare, Chicago, IL, USA, #NA931) was added. After 1 h incubation at RT, the wells were washed three times and bound VHH was detected by addition of 100 µL 0.5 mM 2,2′-azino-bis(3-ethylbenzothiazoline-6-sulfonic acid) (ABTS; Sigma Aldrich, #A1888) in 50 mM citric acid pH 4.0 supplemented with 0.05% (*v*/*v*) H_2_O_2_. The increase in absorbance at 405 nm was measured in a microplate spectrophotometer (Multiskan GO, Thermo Fisher Scientific).

On- and off-rates of the fluorescein-specific VHH were determined by bio-layer interferometry using the Octet^®^ RED96 System (FortéBio, Fremont, CA, USA). The assay temperature was set to 30 °C. Aminopropylsilane (APS) biosensors were equilibrated with PBS and loaded with Flu-BSA or BSA (10 µg/mL in PBS) for 300 s. After blocking with assay buffer (PBS supplemented with 1% (*w*/*v*) BSA) (baseline, 300 s), association of the VHH (1:1 dilution series 0–600 nM in assay buffer) was performed for 300 s, followed by dissociation in assay buffer for 300 s. Control experiments with BSA-coated biosensors showed no association with the highest applied VHH concentration. Assays conducted in the absence of VHH on Flu-BSA-coated biosensors served as reference and were subtracted from the data. Association and dissociation curves were fitted globally according to a 1:1 bimolecular interaction model (Figure 7B). Based on the residuals, curves obtained with 600 nM were excluded. Furthermore, the lowest concentrations (18.8 and 37.5 nM) showed only weak responses and were also excluded.

### 4.5. Synthetic Flu-VHH Library and Phage Display

The synthetic Flu-VHH library was designed by randomizing CDR2 and CDR3 as described in the main text (see Section 2.4 and Figure 5). The library was constructed by polymerase cycling assembly using oligonucleotides containing the corresponding ambiguous codons (oHJW352-oHJW356, listed in Supplementary Table S2). The resulting VHH library was ligated into phagemid pCANTAB6 (*Nco*I/*Not*I), purified via the High Pure PCR Cleanup Micro kit (Roche, #04983955001), followed by transformation of *E. coli* XL-1 blue (Agilent, Santa Clara, CA, USA, #200249) using an electroporator (Multiporator, Eppendorf, Hamburg, Germany) with Gene Pulser^®^ electroporation cuvettes (0.2 cm gap; Bio-Rad, #1652082) at 2.5 kV for 5 ms. Transformed bacteria were grown on LB agar supplemented with 1% (*w*/*v*) glucose and 100 µg/mL ampicillin. A library size of 1.05 × 10^7^ clones was estimated by plating dilutions of each transformation. Phages were produced by inoculating 50 mL 2xYT medium supplemented with 2% (*w*/*v*) glucose and 100 µg/mL ampicillin to an OD_600_ = 0.1. Bacteria were grown at 37 °C and 150 rpm. At OD_600_ = 0.5, the cells were incubated with 2.5 × 10^11^ KM13 helper phages for 30 min at 37 °C without shaking. The medium was exchanged by centrifugation at 3000× *g* for 30 min and resuspending the cell pellet in 500 mL 2xYT supplemented with 100 µg/mL ampicillin and 50 µg/mL kanamycin. Phages were produced at 30 °C and 150 rpm overnight. The phage-displayed VHH library was prepared by pelleting the bacteria at 10,000× *g* and 4 °C for 30 min, mixing 400 mL supernatant with 100 mL phage preparation solution (20% (*w*/*v*) PEG-8000, 2.5 M NaCl) and incubation on ice for 1 h. Phage particles were spun down at 10,000× *g* and 4 °C for 30 min and resuspended in 8 mL PBS. After addition of 2 mL phage preparation solution, the phage suspension was incubated for 30 min on ice and spun down at 10,000× *g* and 4 °C for 30 min. The pellet was resuspended in 4 mL PBS, supplemented with 15% (*v*/*v*) glycerol. Remaining bacterial debris was removed by centrifugation at 10,000× *g* and 4 °C for 10 min. The obtained phage titer was 9 × 10^9^ cfu/µL.

For phage panning, 10^12^/mL phages were incubated with 0.2% (*w*/*v*) BSA for 30 min at RT with rotation. Nunc Maxisorp 96-well plates (VWR, Radnor, PA, USA, #62409-002) were coated with 1 µg/well Flu-BSA or BSA (negative control), blocked with blocking buffer (TBS, supplemented with 0.05% (*v*/*v*) Tween-20 and 1% (*w*/*v*) BSA), washed with TBST (TBS, supplemented with 0.05% (*v*/*v*) Tween-20) and incubated with 100 µL phage suspension for 2 h at RT with agitation. Wells were washed 15 times with TBST. In the first round of panning, phages were eluted by incubation with 100 µL trypsin (0.25 mg/mL) for 30 min. Trypsin was then inactivated with 0.1 mg/mL 4-(2-aminoethyl)benzenesulfonyl fluoride hydrochloride (AEBSF; Carl Roth, Karlsruhe, Germany, #2931). In the second round of panning, phages were competitively eluted, first with 10 µM fluorescein for 30 min, then with 1 mM fluorescein for 1 h. *E. coli* TG1 cells were infected with eluted phages and grown in LB, supplemented with 100 µg/mL ampicillin and 2% (*w*/*v*) glucose at 37 °C and 150 rpm overnight. To estimate the enrichment of phages eluted from the selection wells versus control wells (BSA coating), the phages were titrated. For this, *E. coli* TG1 cells (Lucigen, Middleton, WI, USA, #60502) were infected with dilutions of phages and the number of colonies grown on LB agar supplemented with 100 µg/mL ampicillin and 1 % (*w*/*v*) glucose were determined. The enrichment factor was calculated by dividing the phage titer obtained from the selection wells by the titer obtained from control wells.

ELISA was conducted to screen for fluorescein-binding VHH. For this, single colonies of phage-infected cells were picked and grown in a 96-well plate with 2xYT, supplemented with 100 µg/mL ampicillin, 2% (*w*/*v*) glucose, and 10% (*v*/*v*) glycerol at 37 °C overnight. 4 µL of the overnight cultures were transferred to fresh 2xYT medium, supplemented with 100 µg/mL ampicillin, 0.1% (*w*/*v*) glucose, and incubated at 37 °C with and 200 rpm. After 2 h, 1 mM IPTG was added and the cultures were incubated for additional 5 h at 37 °C and 200 rpm. The cells were pelleted at 3000× *g* for 10 min and the supernatants were removed. Pellets were frozen at −20 °C overnight, thawed and resuspended in PBS. The suspension was centrifuged at 3000× *g* for 10 min and 1:2 dilutions of the supernatants were tested by ELISA.

## Supplementary Materials

Supplementary materials can be found at http://www.mdpi.com/1422-0067/19/11/3444/s1.

## Abbreviations

BoNT/A-LC	Botulinum neurotoxin serotype A light chain
BSA	Bovine serum albumin
CDR	Complementarity-determining region
ELISA	Enzyme-linked immunosorbent assay
EMA	European Medicines Agency
Fab	Fragment antigen-binding
FDA	Food and Drug Administration
Flu	Fluorescein
HCAb	Heavy-chain-only antibody
sdAb	Single-domain antibody
scFv	single-chain variable fragment
VH	Heavy chain variable domain
VHH	variable domain of camelid heavy-chain-only antibodies
VL	Light chain variable domain
VNAR	Variable domain of the immunoglobulin new antigen receptor (IgNAR) from sharks
