# Supplementary material for: A Two-Step Approach for the Design and Generation of Nanobodies
Source: Int J Mol Sci. 2018 Nov 2;19(11):3444. doi: 10.3390/ijms19113444 (PMC6274671; doi:10.3390/ijms19113444)
Supplement: Supplementary file 1 [file ijms-19-03444-s001.pdf]

International Journal of *Molecular Sciences*

MDPI

*Article*

# A two-step approach for the design and generation of nanobodies

## Supplementary Information

Hanna J. Wagner[1,2,3], Sarah Wehrle[1], Etienne Weiss[4], Marco Cavallari[1,3,*], Wilfried Weber[1,2,3,*]

*Correspondence: wilfried.weber@biologie.uni-freiburg.de

marco.cavallari@bioss.uni-freiburg.de

## Contents

**Figure S1.** Production and purification of grafted VHH. 2

**Figure S2.** Production and purification of affinity maturated fluorescein-binding VHH-D4. 2

**Figure S3.** Fluorescein binding capability of the selected VHH-D4. 3

**Table S1.** Plasmids used in this study. 4

**Table S2.** Oligonucleotides used in this study. 6

**References** 7

International Journal of Molecular Sciences
MDPI

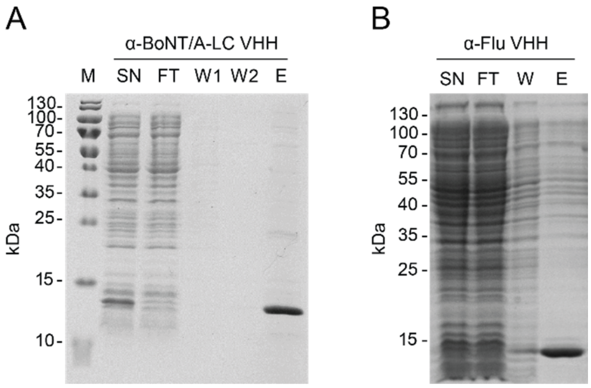


**Figure S1.** Production and purification of grafted VHH. **(a)** SDS-PAGE of the purification of the BoNT/A-LC-binding VHH. The VHH was produced in *E. coli* SHuffle T7 Express and purified via Ni-NTA affinity chromatography. M, molecular weight marker; SN, supernatant (cleared lysate); FT, flow-through; W1, first wash; W2, second wash; E, elution. **(b)** SDS-PAGE of the purification of the fluorescein-binding VHH. The VHH was produced in *E. coli* SHuffle T7 Express and purified via protein A affinity chromatography. SN, supernatant (cleared lysate); FT, flow-through; W, wash; E, elution.

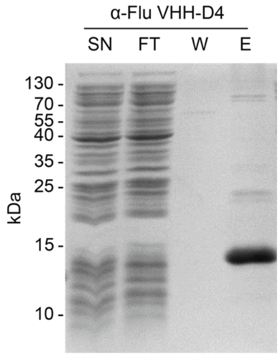


**Figure S2.** Production and purification of affinity maturated fluorescein-binding VHH-D4. The VHH was produced in *E. coli* SHuffle T7 Express and purified via Ni-NTA affinity chromatography. Samples of the cleared lysate (SN, supernatant), flow-through (FT), wash (W), and elution (E) were subjected to SDS-PAGE.

International Journal of
*Molecular Sciences*

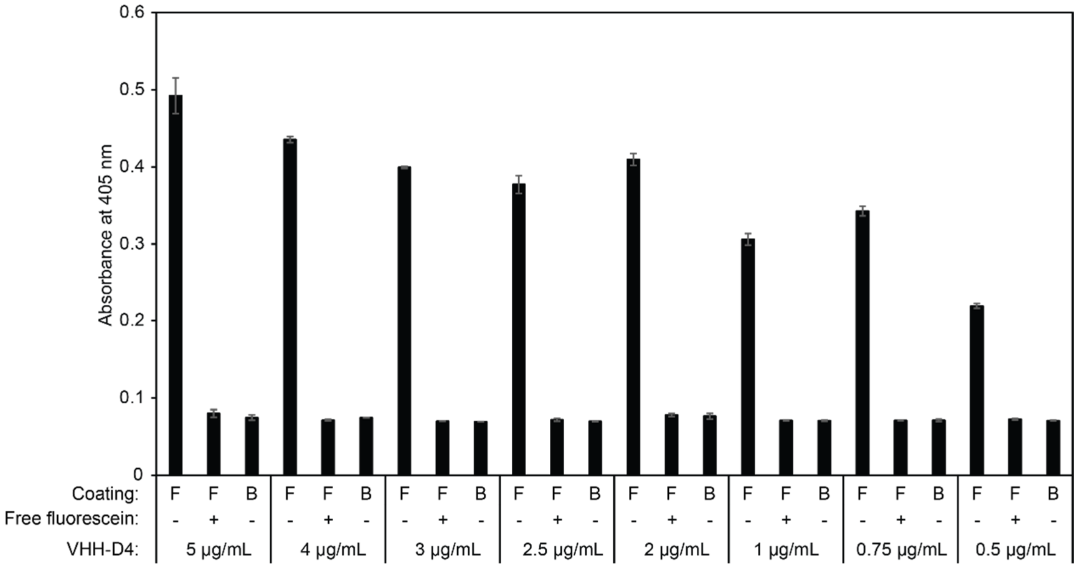


**Figure S3.** Fluorescein binding capability of the selected VHH-D4. Wells were coated with BSA (B) or fluorescein-conjugated BSA (F) and the binding of the indicated concentrations of VHH-D4 were evaluated in the absence (-) or presence (+) of 1 mM free fluorescein. Mean values of three replicates +/- standard deviations are shown.

International Journal of *Molecular Sciences*

MDPI

**Table S1.** Plasmids used in this study.

| Plasmid | Description | Reference |
|---|---|---|
| pHJW202 | $P_{T7}$-Flu-VHH-graft-$His_6$<br>Bacterial expression vector for the production of a fluorescein-binding VHH generated by grafting the CDRs from VH of the fluorescein-binding scFv-E2 [1] onto the framework of the enhancer VHH [2]. The Flu-VHH-graft was ordered as gBlocks Gene Fragment (see below; Integrated DNA Technologies) and cloned into pRSET-mod [3] (*Hind*III/*Nde*I). The cysteine at position 68 (Kabat numbering) was changed to threonine by site-directed mutagenesis using oligonucleotides oHJW350 and oHJW351.<br><br>Flu-VHH-graft gBlocks Gene Fragment: *Nde*I, His-tag, *Hind*III<br>gaaggagatatacatatggcgcaggtgcagctggtggaaagcggcggcgcgctggtgcagccgggcggcagcctgcgcc<br>tgagctgcgcggcgagcggctttacctttggcagctttagcatgagctggtatcgccaggcgaccggcaaagaacgcgaatg<br>ggtggcgggcctgagcgcgcgcagcagcctgacccattatgcggatagcgtgaaaggccgctttaccattagccgcgataac<br>gcgcgcaacaccgtgtatctgcagatgaacagcctgaaaccggaagataccgcggtgtattattgcgcgcgccgcagctatg<br>atagcagcggctatgcgggccatttttatagctatatggatgtgtggggccagggcacccaggtgaccgtgagcagccatcat<br>caccatcatcattaaaagcttgatc | This work |
| pHJW206 | $P_{T7}$-Flu-VHH-D4-$His_6$<br>Bacterial expression vector encoding fluorescein-binding VHH-D4, selected from a synthetic library by phage display. The sequence of VHH-D4 was amplified from phagemid (pCANTAB6) using oHJW377 and oHJW378 and cloned into pRSET-mod [3] (*Hind*III/*Nde*I) via Gibson assembly [4]. | This work |
| pHJW258 | $P_{T7}$-Streptag-BoNT/A-LC<br>Bacterial expression vector encoding the light chain of Botulinum neurotoxin A, fused to Strep-tag® for purification. The BoNT/A-LC sequence was a gift from Axel Brunger (Addgene plasmid #31602) [5]. The sequence (residues 8-415) was amplified with oligonucleotides oHJW167 and oHJW168 and digested with *Kpn*I and *Xho*I. The backbone (pRSET-mod [3]) was amplified with oHJW166 and oHJW171 and digested with *Nde*I and *Xho*I. Both fragments were ligated with annealed oligonucleotides oHJW169 and oHJW170, resulting in a bacterial expression vector for His- and S-tagged BoNTA/-LC(8-415) (pHJW62). Amino acids 1-8 and 416-425 were incorporated into pHJW62 by amplifying the BoNT/A-LC and the backbone with oligonucleotide pair oHJW416/oHJW417 and oHJW418/oHJW419, respectively, and assembling both fragments via Gibson cloning, resulting in a bacterial expression vector for His- and S-tagged full-length BoNT/A-LC (pHJW144). Removal of His-tags and insertion of Strep-tag® was conducted by amplifying pHJW144 with oHJW519/oHJW520 and oHJW521/oHJW516, and assembling both fragments by Gibson cloning. | This work |

International Journal of Molecular Sciences

| Plasmid | Description | Reference |
|---|---|---|
| pHJW268 | $P_{T7}$-BoNT/A-LC-VHH-graft-$His_6$<br>Bacterial expression vector encoding a BoNT/A-LC-binding VHH generated by grafting CDRs from VH of the BoNT/A-LC-binding scFv (GenBank accession no. FJ643069, [6]) onto the framework of cAbBCII10 [7]. The VHH sequence was assembled by polymerase cycling assembly using oligonucleotides oHJW594, oHJW595, oHJW596, and oHJW597. The backbone (pRSET-mod [3]) was amplified with oHJW588 and oHJW589. Both fragments were assembled by Gibson cloning. | This work |

International Journal of *Molecular Sciences*

MDPI

**Table S2.** Oligonucleotides used in this study. Annealing sequences are underlined. Ambiguous codons are in bold.

| Oligo | Sequence (5′ → 3′) | Reference |
|---|---|---|
| oHJW166 | CATCATCACCATCACCATTAAAAGCTTCTCGAGGATCCGGCTGCTAACAAAGC | This work |
| oHJW167 | CGGGGTACCGACGACGACGACAAGGCCATGGCTTATAAAGATCCTGTAAATGGTGTTGATATTG | This work |
| oHJW168 | tGTCCGCTCGAGTTAATGGTGATGGTGATGATGCTCCAGTAGTTTAGTAAAATTCATATTATTAATTTCTGTATTTTGACC | This work |
| oHJW169 | TATGCACCATCATCATCATCATTCTTCTGGTCTGGTGCCACGCGGTTCTGGTATGAAAGAAACCGCTGCTGCTAAATTCGAACGCCAGCACATGGACAGCCCAGATCTGGGTAC | This work |
| oHJW170 | CCAGATCTGGGCTGTCCATGTGCTGGCGTTCGAATTTAGCAGCAGCGGTTTCTTTCATACCAGAACCGCGTGGCACCAGACCAGAAGAATGATGATGATGATGGTGCA | This work |
| oHJW171 | GGAATTCCATATGTATATCTCCTTCTTAAAGTTAAAC | This work |
| oHJW350 | CGTGAAAGGCCGCTTTACCATTAGCCGCGATAACG | This work |
| oHJW351 | CGTTATCGCGGCTAATGGTAAAGCGGCCTTTCACG | This work |
| oHJW352 | CGTACCATGGCGCAGGTGCAGC | This work |
| oHJW353 | CCTTTCACGCTATCCGCATA**GNN**GGT**SRA**GCTGCT**GSBGSY**GCTCAGGCCCGCCAC | This work |
| oHJW354 | TATGCGGATAGCGTGAAAGGC | This work |
| oHJW355 | GCTGCTCACGGTCACCTGGGTGCCCTGGCCCCA**AWM**ATC**MAWGNNGKHGKHAANAKR**GCCCGC**GKH**GCCGCTGCT**GKHGKHGKH**GCGGCGCGCAATAATAC | This work |
| oHJW356 | ATAGTTTAGCGGCCGCGCTGCTCACGGTCACCT | This work |
| oHJW377 | CTTTAAGAAGGAGATATACATATGGCGCAGGTGCAGCTG | This work |
| oHJW378 | GTTAGCAGCCGGATCAAGCTTTTAATGATGATGGTGATGATGGCTGCTCACGGTCACCTG | This work |
| oHJW416 | CGACGACAAGGCCATGGCTCCGTTTGTGAACAAACAGTTTAACTATAAAGATCCTGTAAATGGTGTTGATATTGC | This work |
| oHJW417 | CGAGTTAATGGTGATGGTGATGATGAAATTCAAACAGGCCGGTAAAGTTTTTTAGTTTAGTAAAATTCATATTATTAATTTCTGTATTTTGACC | This work |
| oHJW418 | CATCATCACCATCACCATTAACTCG | This work |
| oHJW419 | AGCCATGGCCTTGTCGTC | This work |

| Oligo | Sequence (5′ → 3′) | Reference |
|---|---|---|
| oHJW516 | GATCCACCGCCAGAACCTCCACCTTTTTCGAACTGCGGGTGGCTCCAAGCGCTCCCCATATGTATATCTCCTTCTTAAAGTTAAACAAAATTATTTC | This work |
| oHJW519 | TGGAGCCACCCGCAGTTCGAGAAATCGGCGAAAGAAACCGCTGCTGCTAAATTCGAACGCCAGCACATGGACAGCGCCATGGCTCCGTTTGTG | This work |
| oHJW520 | GCAGCCGGATCCTCGAGTTAAAATTCAAACAGGCCGGTAAAG | This work |
| oHJW521 | TAACTCGAGGATCCGGCTG | This work |
| oHJW588 | CTAAAGCCGCTCGCGGTGCAGCTCAGGCGCAGGCTGCCGCCCGCCTGCACGCTGCCGCCGCCGCTTTCCAC | This work |
| oHJW589 | GGGCCAGGGCACCCAG | This work |
| oHJW594 | CGCGAGCGGCTTTAGCCGCGAATATGGCGTGGGCTGGTTTCGCCAGGCGCCGGGCCAGGAACGCGAATGG | This work |
| oHJW595 | GCGGCTTTTCAGCGCCGGGTTATGCAGGGTATAGCCGCTATCATCAATGCGGCCCAGCCATTCGCGTTCCTGGC | This work |
| oHJW596 | CGGCGCTGAAAAGCCGCTTTACCATTAGCCGCGATATTGCGAAAAACACCGTGACCCTGCAGATGAACAACCTGAAACCGGAAGATACCGCG | This work |
| oHJW597 | CTGGGTGCCCTGGCCCCACACATCAATATCGCCCAGCCACGCGGTGCCGCGGCTCGCATACACGCAATAATAAATCGCGGTATCTTCCGGTTTC | This work |

## References


1. Vaughan, T. J.; Williams, A. J.; Pritchard, K.; Osbourn, J. K.; Pope, A. R.; Earnshaw, J. C.; McCafferty, J.; Hodits, R. A.; Wilton, J.; Johnson, K. S. Human Antibodies with Sub-nanomolar Affinities Isolated from a Large Non-immunized Phage Display Library. *Nat. Biotechnol.* **1996**, *14*, 309–314, doi:10.1038/nbt0396-309.
2. Kirchhofer, A.; Helma, J.; Schmidthals, K.; Frauer, C.; Cui, S.; Karcher, A.; Pellis, M.; Muyldermans, S.; Casas-Delucchi, C. S.; Cardoso, M. C.; Leonhardt, H.; Hopfner, K. P.; Rothbauer, U. Modulation of protein properties in living cells using nanobodies. *Nat. Struct. Mol. Biol.* **2010**, *17*, 133–139, doi:10.1038/nsmb.1727.
3. Zisch, A. H.; Schenk, U.; Schense, J. C.; Sakiyama-Elbert, S. E.; Hubbell, J. A. Covalently conjugated VEGF–fibrin matrices for endothelialization. *J. Control. Release* **2001**, *72*, 101–113, doi:10.1016/S0168-3659(01)00266-8.
4. Gibson, D. G.; Young, L.; Chuang, R.; Venter, J. C.; Hutchison, C. A.; Smith, H. O. Enzymatic assembly of DNA molecules up to several hundred kilobases. *Nat. Methods* **2009**, *6*, 343–5, doi:10.1038/nmeth.1318.
5. Zuniga, J. E.; Schmidt, J. J.; Fenn, T.; Burnett, J. C.; Araç, D.; Gussio, R.; Stafford, R. G.; Badie, S. S.; Bavari, S.; Brunger, A. T. A Potent Peptidomimetic Inhibitor of Botulinum Neurotoxin Serotype A Has a Very Different Conformation than SNAP-25 Substrate. *Structure* **2008**, *16*, 1588–1597, doi:10.1016/j.str.2008.07.011.
6. Sepulveda, J.; Mukherjee, J.; Tzipori, S.; Simpson, L. L.; Shoemaker, C. B. Efficient Serum Clearance of Botulinum Neurotoxin Achieved Using a Pool of Small Antitoxin Binding Agents. *Infect. Immun.* **2010**, *78*, 756–763, doi:10.1128/IAI.01084-09.
7. Vincke, C.; Loris, R.; Saerens, D.; Martinez-Rodriguez, S.; Muyldermans, S.; Conrath, K. General Strategy to Humanize a Camelid Single-domain Antibody and Identification of a Universal Humanized Nanobody Scaffold. *J. Biol. Chem.* **2009**, *284*, 3273–3284, doi:10.1074/jbc.M806889200.
